# Research on Fast Time–Frequency Reconstruction Algorithm for Wideband Compressive Spectrum Sensing

**DOI:** 10.3390/s25061795

**Published:** 2025-03-13

**Authors:** Rangang Zhu, Ce Li, Yanhua Wu, Ruochen Wu, Zhengkun Zhang, Zunhui Wang, Yuliang Lu

**Affiliations:** 1 College of Electronic Engineering, National University of Defense Technology, Hefei 230037, China; zhurangang17@nudt.edu.cn (R.Z.); lice22@nudt.edu.cn (C.L.); zzk20011119@163.com (Z.Z.); 18686332939@163.com (Z.W.); 18214880348@163.com (Y.L.); 2 Anhui Provincial Key Laboratory of Electronic Restriction, Hefei 230037, China; 3 School of Automation, Southeast University, Nanjing 210000, China; 213212254@seu.edu.cn

**Keywords:** wideband spectrum sensing, compressed sensing, compressed power-spectrum estimation, multi-coset sampling, time–frequency reconstruction

## Abstract

Cognitive Radio (CR) is widely acknowledged as a pivotal technology for mitigating the scarcity of spectrum resources, with Transform Domain Communication Systems (TDCSs) regarded as one of the primary candidate technologies for CR. However, conventional Wideband Spectrum Sensing (WBSS) techniques utilized in TDCS exhibit limitations and are insufficient for adapting to the current complex electromagnetic environment. This paper tackles the time–frequency reconstruction challenge in WBSS by proposing a fast time–frequency reconstruction (FTFR) algorithm. The proposed algorithm acquires sub-Nyquist samples through the introduction of a Multi-Coset Sampling structure and reconstructs the autocorrelation of signals across various windows through a series of low-complexity operations. It captures the dynamic variations of signals by integrating spectra from adjacent time windows. In comparison to existing time–frequency reconstruction algorithms in WBSS, the proposed algorithm demonstrates reduced computational complexity. Simulation experiments indicate that the FTFR algorithm can effectively reconstruct the time–frequency characteristics of signals and significantly restore the primary temporal and frequency distributions, even in low Signal-to-Noise Ratio (SNR) environments.

## 1. Introduction

With the advancement of wireless communication technologies, the scarcity of spectrum resources has emerged as a critical constraint limiting the performance improvement of wireless communication systems. Consequently, Cognitive Radio (CR)—a technology capable of dynamically sensing and utilizing spectrum resources—has garnered considerable attention [1]. CR enhances spectral efficiency by adaptively learning from the communication system to sense the spectrum usage in the surrounding environment, fully exploiting spectral holes, and dynamically adjusting its operating frequency to minimize conflicts as much as possible [2]. Transform Domain Communication Systems (TDCS) are regarded as highly promising candidate technologies within the realm of cognitive radio, owing to their flexible spectrum utilization, robust interference resistance, and low probability of being intercepted [3]. To date, the majority of research on spectrum sensing has primarily concentrated on Narrowband Spectrum Sensing (NBSS). However, since TDCS operates over a wide band, NBSS proves inadequate for addressing the inherent complexity and dynamism of wideband signals; thus, it fails to achieve higher throughput or satisfy the demands of contemporary broadband communication systems [4]. Furthermore, TDCS continues to utilize traditional power-spectrum estimation methods for spectrum sensing, including AR spectrum estimation, which fail to account for the increasingly complex variations in the electromagnetic environment. Consequently, research into Wideband Spectrum Sensing (WBSS) that is suitable for TDCS holds substantial practical significance.

Transitioning from Narrowband Spectrum Sensing (NBSS) to Wideband Spectrum Sensing (WBSS), an intuitive approach involves dividing the entire wideband into multiple narrowbands and applying NBSS methods to sequentially sense each sub-band, thereby achieving comprehensive sensing across the broadband. However, this method presents significant drawbacks in practical applications: it results in high latency, fails to meet real-time requirements, and operates inefficiently [5]. An alternative solution, as proposed in [6,7], employs a multi-resolution sensing strategy that optimizes detection accuracy through the adjustment of wavelet-transform basis function precision. This process occurs in two stages: initially, a coarse-grained detection is performed across the entire band to filter out several potentially active narrowbands, followed by fine-grained detection focused exclusively on these promising narrowbands to minimize sensing costs. However, as the spectrum range that requires detection continues to expand, the initial detection may characterize each narrowband as potentially active, necessitating sequential fine-grained detection of every narrowband, which inevitably results in substantial increases in both time and energy consumption.

According to the Nyquist sampling theorem, conventional Wideband Spectrum Sensing (WBSS) methods are constrained by the sampling rate of the front-end Analog-to-Digital Converter (ADC) when the spectrum width that requires detection is substantial. This situation creates a dilemma, if the sampling rate requirements are satisfied, the costs associated with hardware, energy consumption, and scalable deployment may become excessively high, rendering large-scale deployment unfeasible. Conversely, if these costs are controlled, meeting the real-time sensing requirements becomes impractical. In this context, the advent of Compressed Sensing (CS) technology [8] presents a novel solution for WBSS. According to the theory of CS, if a signal exhibits sparse or compressible characteristics, it can be efficiently reconstructed from a limited number of random measurements, thereby achieving low-dimensional representation and a high probability of recovery of the original signal [9,10]. A substantial number of scholars have researched and developed various sub-Nyquist-based spectrum sensing algorithms grounded in CS principles. Based on the nature of the information extracted from sub-Nyquist samples, these methods can primarily be categorized into two categories: CS-based approaches and compressive covariance sensing (CCS)-based approaches.

In CS-based approaches, the essence lies in fully exploiting the sparsity of signals within the frequency domain, acquiring corresponding data samples through sub-Nyquist sampling, and subsequently reconstructing the wideband analog signal using various reconstruction algorithms [11,12]. Although the application of CS technology can indeed reduce the sampling rate and provide certain advantages for WBSS, complex reconstruction algorithms have emerged as a bottleneck in enhancing real-time processing capabilities. To address this impasse, studies [13,14] have developed algorithms that simplify signal reconstruction without necessitating additional samples, thereby providing new directions for future research. Reference [15] utilizes model selection methods to estimate the sparsity of the signal, thereby eliminating reliance on prior knowledge of signal sparsity as configured in earlier research, thus enhancing the algorithm’s adaptability and accuracy. Reference [16] proposes a rapid compressive Wideband Spectrum Sensing method that further reduces computational complexity while preserving high-precision spectrum reconstruction performance through the joint utilization of low-rank and sparse properties. This method operates without the necessity for prior knowledge of sparsity, rendering it compatible with low-complexity platforms for real-time sub-Nyquist rate sensing.

When applying compressive sensing to reconstruct wideband analog signals, several challenges can arise, including the difficulty of achieving a sparse representation of actual signals, high computational complexity, noise interference that degrades the quality of the reconstructed signals and more. To address these challenges, numerous scholars have undertaken a series of in-depth studies. Dyonisius Dony Ariananda was one of the first researchers to propose a wideband power-spectrum estimation algorithm grounded in compressive sampling [17]. This algorithm does not enforce sparsity constraints on the power spectrum and can effectively recover the power spectrum of wideband sensed signals from sub-Nyquist rate samples, eliminating the necessity to reconstruct the original signal. Subsequently, reference [18] employed the asymptotic theory of circulant matrices to introduce a novel dimensionality reduction technique that simplifies existing structured covariance estimation algorithms, achieving comparable performance at a reduced computational cost. Reference [19] leverages the statistical structure of stochastic processes for signal compression, thereby providing an alternative perspective for inference that is independent of sparsity. Another approach involves utilizing existing sampling structures to directly reconstruct the power spectrum of wideband analog signals from sub-Nyquist samples through optimized algorithms [20,21,22,23,24]. This can significantly reduce the computational burden of power-spectrum reconstruction while providing new insights for the design of fast time–frequency reconstruction algorithms in this study.

In practical applications, such as wireless communication, electronic warfare, and radar, traditional spectrum sensing and time–frequency reconstruction schemes often have limitations. On the one hand, traditional methods usually focus on static power-spectrum analysis and have difficulty effectively capturing the dynamic changes of signals over time. This results in an inability to timely and accurately sense critical information like spectrum holes in rapidly changing signal environments. On the other hand, despite research by many scholars on the time–frequency reconstruction of wideband signals [25,26,27,28], existing algorithms commonly struggle with real-time sensing. Further analysis reveals two main reasons for this issue: first, some algorithms lose temporal information during processing, preventing a complete and accurate representation of the signal’s time–frequency characteristics; second, some algorithms retain temporal information but analyze multiple short time segments, which not only reduces accuracy but also significantly increases computational load, severely limiting real-time sensing capabilities. To address these shortcomings of traditional schemes and inspired by fast compressive covariance sensing [20], this paper proposes a fast time–frequency reconstruction algorithm. It aims to effectively solve the real-time sensing problems of existing algorithms and provide a more efficient and accurate solution for the time–frequency reconstruction of wideband signals. This will better meet the needs of signal dynamic-change monitoring and spectrum resource management in practical applications.

The remainder of this paper is organized as follows. Section 2 summarizes the WBSS model and the fundamental principles of wideband compressive covariance sensing. Section 3 focuses on the proposed FTFR algorithm, detailing its procedure and analyzing its computational complexity. Section 4 presents experimental validations of the algorithm’s reconstruction performance. Finally, Section 5 concludes the paper with a summary.

## 2. Principles of Compressed Spectrum Sensing

In this section, we explore the principles of wideband compressive covariance sensing. We begin by presenting the model of Wideband Spectrum Sensing to provide a comprehensive review of its theoretical foundations. This is succeeded by a concise overview of existing algorithms for wideband compressive power-spectrum estimation.

### 2.1. Wideband Compressive Spectrum Sensing Model

In wireless communication environments, wideband signals generally exhibit spectral sparsity, indicating that within the entire frequency band only a limited number of sub-bands contain non-zero power due to signal activity. Building upon this concept, wideband compressive spectrum sensing harnesses the theory of CS, effectively exploiting signal sparsity to significantly reduce the sampling rate requirements for wideband signals while preserving the accuracy of spectrum sensing. Subsequently, we systematically review the comprehensive process of wideband compressive spectrum sensing.

**Figure 1:**
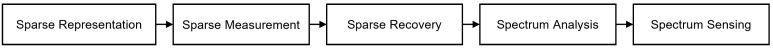
Block diagram of wideband compressive spectrum sensing.

As illustrated in Figure 1, the process of wideband compressive spectrum sensing primarily consists of five steps: sparse representation, sparse measurement, sparse reconstruction, spectrum analysis, and spectrum sensing.

1. Sparse Representation: Let us consider a signal *x* containing *N* observations that demonstrates sparsity in a specific domain, with a sparsity level of K≪N. If the signal does not exhibit sparsity in the designated domain, an appropriate transformation basis can be selected for projection, thereby rendering the signal sparse in the transformed domain. This process can be mathematically represented by Equation (1). In this context, φ represents the transformation basis; if *x* is already sparse, then φ becomes the identity matrix $I_{N}$.
  (1) s=φ×x
  In this equation, *s* represents the sparse representation of the signal *x* within the transformed domain.
2. Sparse Measurement: Upon completion of the sparse representation, the sparse signal is multiplied by a measurement matrix Φ of size M×N, where the number of measurements *M* is associated with the sparsity level, the properties of the measurement matrix, and the chosen reconstruction method, with M<N. The resulting measurement vector is given by Equation (2)
  (2) $$\begin{array}{cc} y & =\Phi \times s\\ \; & =\Phi \times \varphi \times x \end{array}$$
  In this context, *y* represents the compressed measurements obtained from the sparse signal *s*, and Φ denotes the measurement matrix used for acquiring these measurements. To realize this process, various sampling structures have been proposed, including the Analog-to-Information Converter (AIC) [17], Modulated Wideband Converter (MWC) [29], and Multi-Coset Sampling (MCS) [30]. These structures can effectively reduce the number of samples required while preserving the key features of the signal.
3. Sparse Reconstruction: The low-dimensional signal obtained from sparse measurements cannot be directly utilized for spectrum analysis; consequently, it is essential to employ reconstruction algorithms to approximate the original sparse spectrum signal. Commonly employed reconstruction algorithms include Basis Pursuit (BP) [8], Orthogonal Matching Pursuit (OMP) [14], Compressive Sampling Matching Pursuit (CoSaMP) [31], and Simultaneous Orthogonal Matching Pursuit (SOMP) [32]. These algorithms are fundamentally grounded in optimization theory and rely on the sparsity of the signal to accurately reconstruct the original spectral information.
4. Spectrum Analysis: After obtaining the reconstructed signal, techniques such as the Fast Fourier Transform (FFT) can be employed to transform the signal into the frequency domain, facilitating the analysis of its spectral characteristics, which provides the foundation for spectrum sensing.
5. Spectrum Sensing: Finally, in the frequency domain, unoccupied spectrum bands, referred to as spectrum holes, can be identified by establishing an energy threshold. This step typically utilizes energy detection [5], which entails scanning the reconstructed spectrum to identify regions where the energy falls below the established threshold, thereby indicating potential available spectrum resources.

### 2.2. Wideband Compressive Power-Spectrum Estimation

In 2012, Dr. Geert Leus and his research team were among the pioneers in proposing wideband compressive power-spectrum estimation [17]. This method establishes the autocorrelation relationship between sub-Nyquist samples and Nyquist-rate samples through periodic sampling processes in both the time and frequency domains. By not imposing any sparsity constraints, it leverages the cross-correlations of the compressed signal and utilizes simple least squares to reconstruct the signal’s autocorrelation, from which the power spectrum is subsequently calculated. The algorithm for reconstructing the power spectrum in the time domain has significantly informed our research efforts. A brief overview of this algorithm is presented in the following section.

**Figure 2:**
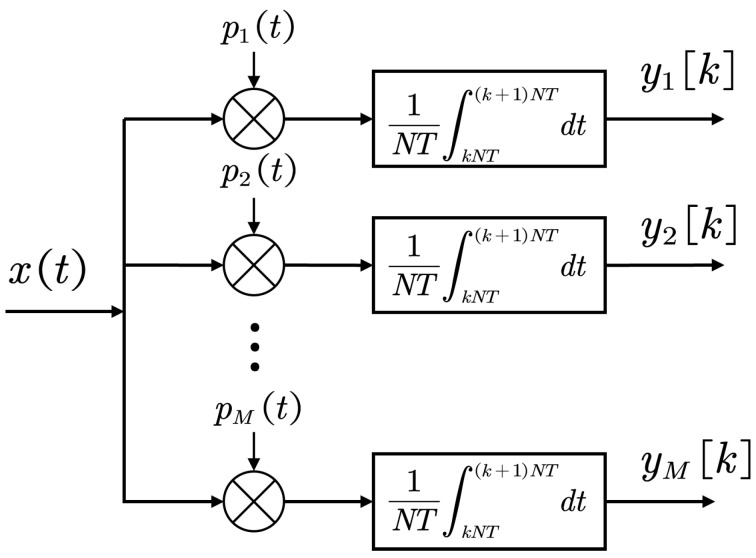
The architecture of the analog to information converter (AIC).

Consider a wide-sense stationary analog signal $x\left(t\right)$ with a bandwidth of $\frac{1}{T}$.The signal $x\left(t\right)$ is sampled below the Nyquist rate using AIC, and the structure of AIC is shown in Figure 2. AIC is a sampling structure with M channels. In the *i*-th channel, the signal $x\left(t\right)$ is modulated by a complex-valued periodic waveform $p_{i}\left(t\right)$ with period NT. In practice, $p_{i}\left(t\right)$ can be chosen as a periodic square wave:

(3) $$p_{i}\left(t\right)=\mathrm{sign}\left(\cos\left(2\pi f_{i}t\right)\right)$$

where $f_{i}$ denotes the modulation frequency of the *i*-th channel. Alternatively, a random modulation approach can be employed, generating $p_{i}\left(t\right)$ according to a specific probability distribution, such as complex Gaussian sampling or randomly selecting values from the set $\left\{-1,1\right\}$ for binary sampling. After modulation by the complex periodic waveform $p_{i}\left(t\right)$, the signal is integrated by an integrator with period NT. Obviously, the sampling rate of AIC is $\frac{1}{N}$ times that of the Nyquist sampling rate. The output at the *k*-th sampling index of the *i*-th channel can be expressed as:

(4) $$\begin{array}{cc} y_{i}\left[k\right] & =\frac{1}{NT}\int_{kNT}^{(k+1)NT}p_{i}\left(t\right)x\left(t\right)\;dt\\ \; & =\frac{1}{T}\int_{kNT}^{(k+1)NT}c_{i}\left(t-kNT\right)x\left(t\right)\;dt \end{array}$$

where $c_{i}\left(t\right)$ denotes one period of $\frac{1}{N}p_{i}\left(t\right)$, that is

(5) $$c_{i}\left(t\right)=\left\{\begin{array}{c} \frac{1}{N}p_{i}\left(t\right),\;\;\;\;0\leq t<NT\\ 0\;\;\;\;\;\;\;\;\;\;\;\;\;\;,\;\;\;\;\mathrm{otherwise} \end{array}\right.$$

Assume that $c_{i}\left(t\right)$ is a piecewise constant function, with constant values in each interval of length *T*, i.e., $c_{i}\left(t\right)=c_{i}\left[-n\right]$; for $nT\leq t\leq \left(n+1\right)T$, we have:

(6) $$\begin{array}{cc} y_{i}\left[k\right] & =\sum_{n=0}^{N-1}c_{i}\left[-n\right]\frac{1}{T}\int_{(kN+n)T}^{(kN+n+1)T}x\left(t\right)dt\\ \; & =\sum_{n=0}^{N-1}c_{i}\left[-n\right]x\left[kN+n\right]\\ \; & =\sum_{n=1-N}^{0}c_{i}\left[n\right]x\left[kN-n\right] \end{array}$$

**Figure 3:**
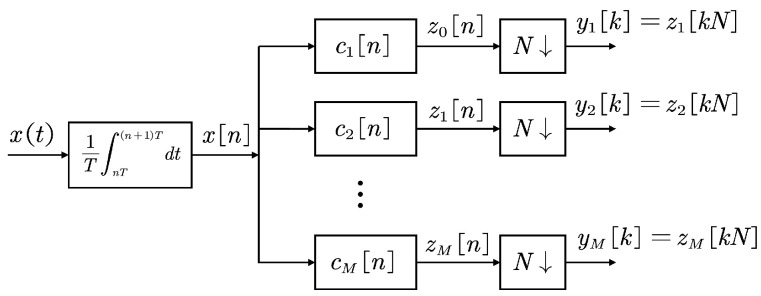
A digital interpretation of the architecture of the analog to information converter (AIC).

Figure 3 illustrates the digital interpretation of the AIC sampling device. The process begins with an integrator that produces $x\left[n\right]$, followed by a set of channels. In the *i*-th channel, filtering is performed by a filter $c_{i}\left[n\right]$ of length *N*, and then *N*-times decimated to obtain the final sampled result, i.e., $y_{i}\left[k\right]=z_{i}\left[kN\right]$, where

(7) $$\begin{array}{cc} z_{i}\left[n\right] & =c_{i}\left[n\right]*x\left[n\right]\\ \; & =\sum_{m=1-N}^{0}c_{i}\left[m\right]x\left[n-m\right] \end{array}$$

* denotes the convolution operator. This completes the compressed sampling operation of the wide-sense stationary analog signal $x\left(t\right)$. Next, the autocorrelation $r_{x}\left[k\right]$ of the original signal is reconstructed using the cross-correlation $r_{y_{i},y_{j}}\left[k\right]$ between $y_{i}\left[k\right]$ and $y_{j}\left[k\right]$.

According to the definition of cross-correlation and the relationship $y_{i}\left[k\right]=z_{i}\left[kN\right]$, the cross-correlation between $y_{i}\left[k\right]$ and $y_{j}\left[k\right]$ can be expressed as a *N*-times sampled version of the cross-correlation function between $z_{i}\left[k\right]$ and $z_{j}\left[k\right]$, given by:

(8) $$\begin{array}{cc} r_{y_{i},y_{j}}\left[k\right] & =E\left[y_{i}\left[l\right]y_{j}*\left[l-k\right]\right]\\ \; & =E\left[z_{i}\left[lN\right]z_{j}*\left[\left(l-k\right)N\right]\right]\\ & =r_{z_{i},z_{j}}\left[kN\right] \end{array}$$

where E represents statistical expectation, which is used to describe the mean characteristics of random signals. Substituting expression (7) into expression (8), $r_{z_{i},z_{j}}\left[n\right]$ can be written as:

(9) $$\begin{array}{cc} r_{z_{i},z_{j}}\left[n\right] & =r_{c_{i},c_{j}}\left[n\right]*r_{x}\left[n\right]\\ & =\sum_{m=-N+1}^{N-1}r_{c_{i},c_{j}}\left[m\right]r_{x}\left[n-m\right] \end{array}$$

where

(10) $$\begin{array}{cc} r_{c_{i},c_{j}} & =c_{i}\left[n\right]*c_{j}^{*}\left[-n\right]\\ \; & =\sum_{m=1-N}^{0}c_{i}\left[m\right]c_{j}^{*}\left[m-n\right] \end{array}$$

represents the deterministic cross-correlation function between $c_{i}\left[n\right]$ and $c_{j}\left[n\right]$. Combining expressions (8)–(10), we obtain:

(11) $$\begin{array}{cc} r_{y_{i},y_{j}}\left[k\right] & =r_{z_{i},z_{j}}\left[kN\right]\\ \; & =\sum_{m=-N+1}^{N-1}r_{c_{i},c_{j}}\left[m\right]r_{x}\left[kN-m\right]\\ \; & =\sum_{l=0}^{1}r_{c_{i},c_{j}}^{T}\left[l\right]r_{x}\left[k-l\right] \end{array}$$

where

(12) $$r_{c_{i},c_{j}}\left[0\right]={\left[r_{c_{i},c_{j}}\left[0\right],r_{c_{i},c_{j}}\left[1\right],\cdots ,r_{c_{i},c_{j}}\left[-N+1\right]\right]}^{T}$$

(13) $$r_{c_{i},c_{j}}\left[1\right]={\left[r_{c_{i},c_{j}}\left[N\right],r_{c_{i},c_{j}}\left[N-1\right],\cdots ,r_{c_{i},c_{j}}\left[1\right]\right]}^{T}$$

(14) $$r_{x}\left[k\right]={\left[r_{x}\left[kN\right],r_{x}\left[kN+1\right],\cdots ,r_{x}\left[\left(k+1\right)N-1\right]\right]}^{T}$$

Concatenating $M^{2}$ distinct cross-correlation functions $r_{y_{i},y_{j}}\left[k\right]$ forms the vector $r_{y}\left[k\right]$, expressed as:

(15) $$r_{y}\left[k\right]=\sum_{l=0}^{1}R_{c}\left[l\right]r_{x}\left[k-l\right]$$

Here, $R_{c}\left[0\right]$ and $R_{c}\left[1\right]$ are $M^{2}\times N$ matrices, given by:

(16) $$R_{c}\left[0\right]={\left[\cdots ,r_{c_{i},c_{j}}\left[0\right],\cdots \right]}^{T}$$

(17) $$R_{c}\left[1\right]={\left[\cdots ,r_{c_{i},c_{j}}\left[1\right],\cdots \right]}^{T}$$

Due to the band-limited nature of $x\left[n\right]$, the range of $r_{y}\left[k\right]$ is restricted to −L≤k≤L, where *L* can be set according to practical needs. The expressions for $r_{x}\left[k\right]$ and $r_{y}\left[k\right]$ are shown in Equations (18) and (19), respectively.

(18) $$r_{x}={\left[r_{x}^{T}\left[0\right],r_{x}^{T}\left[1\right],\cdots ,r_{x}^{T}\left[L\right],r_{x}^{T}\left[-L\right],\cdots ,r_{x}^{T}\left[-1\right]\right]}^{T}$$

(19) $$r_{y}={\left[r_{y}^{T}\left[0\right],r_{y}^{T}\left[1\right],\cdots ,r_{y}^{T}\left[L\right],r_{y}^{T}\left[-L\right],\cdots ,r_{y}^{T}\left[-1\right]\right]}^{T}$$

By examining the ranges of $r_{x}\left[k\right]$ and $R_{c}\left[1\right]$, Equation (11) can be expressed as a cyclic convolution, leading to the vector form:

(20) $$r_{y}=R_{c}r_{x}$$

Here, $R_{c}$ is a $\left(2L+1\right)M^{2}\times \left(2L+1\right)N$ fast cyclic matrix given by:

(21) $$R_{c}=\left[\begin{array}{ccccc} lR_{c}\left[0\right] & & & & R_{c}\left[1\right]\\ R_{c}\left[1\right] & R_{c}\left[0\right] & & & \\ \; & R_{c}\left[1\right] & R_{c}\left[0\right] & & \\ \; & & \ddots & \ddots & \\ \; & & & R_{c}\left[1\right] & R_{c}\left[0\right] \end{array}\right]$$

where

(22) $$R_{c}\left[0\right]={\left[\cdots ,r_{c_{i},c_{j}}\left[0\right],\cdots \right]}^{T}$$

(23) $$R_{c}\left[1\right]={\left[\cdots ,r_{c_{i},c_{j}}\left[1\right],\cdots \right]}^{T}$$

If $R_{c}$ is a full-rank matrix and $M^{2}\geq N$, Equation (20) can be theoretically solved using the least-squares method. However, due to the potential ill-conditioning of $R_{c}$, direct least squares may amplify measurement noise and lead to error accumulation. To mitigate this, we introduce the Tikhonov regularization approach:

(24) $${\hat{r}}_{x}=\underset{r_{x}}{\arg\min}{∥r_{y}-R_{c}r_{x}∥}^{2}-\lambda {∥r_{x}∥}^{2}$$

where λ>0 is a regularization parameter selected via the *L*-curve criterion to balance data fidelity and solution smoothness.

Furthermore, the solution to the matrix Equation (20) is complicated by the error-accumulating nature of the special matrix $R_{c}$ in $r_{y}$. This may introduce extra error and complicates the solution. To address this, in addition to adopting the Tikhonov regularization approach, another strategy involves considering the structural characteristics of $R_{c}$ into a diagonal block matrix $Q_{c}$, which is expressed as:

(25) $$Q_{c}\left(\omega \right)=\sum_{k=0}^{1}R_{c}\left[k\right]e^{-jk\omega }$$

We define the vectors $q_{x}$ and $q_{y}$ as the Discrete Fourier Transform (DFT) of $r_{x}$ and $r_{y}$, respectively, as shown below:

(26) $$q_{x}=\left(F_{2L+1}⊗I_{N}\right)r_{x}$$

(27) $$q_{y}=\left(F_{2L+1}⊗I_{M^{2}}\right)r_{y}$$

Based on these definitions, Equation (20) can be reformulated as:

(28) $$q_{y}=Q_{c}q_{x}$$

By expressing $q_{x}$ and $q_{y}$ as frequency components, Equation (28) can be rewritten as a set of matrix equations, as shown in Equation (29):

(29) $$q_{y}\left(2\pi \frac{l}{2L+1}\right)=Q_{c}\left(2\pi \frac{l}{2L+1}\right)q_{x}\left(2\pi \frac{l}{2L+1}\right)$$

If $Q_{c}$ is full-rank for all *l*, then $q_{x}$ can be solved using the least-squares method. Subsequently, $r_{x}$ can be reconstructed via the Inverse Discrete Fourier Transform (IDFT), and finally, the power spectrum of the wide-sense stationary analog signal $x\left(t\right)$ can be reconstructed by performing a DFT on $r_{x}$.

## 3. Fast Time–Frequency Reconstruction Algorithm

In Wideband Spectrum Sensing, capturing the dynamic changes of signals over time is a key challenge. Traditional methods often focus on static power-spectrum analysis, which fails to effectively capture these dynamic changes. This can lead to misjudgments in complex electromagnetic environments. To address this, inspired by the efficient power-spectrum reconstruction algorithms utilizing Multi-Coset Sampling (MCS) referenced in [17,20], this paper introduces an effective time–frequency analysis algorithm. The core of this algorithm resides in the integration of sub-Nyquist sampling technology for signal compression, along with time-window segmentation and fast autocorrelation estimation, which facilitates the rapid construction of the time–frequency distribution in both the time and frequency domains. By reconstructing the time–frequency map, the algorithm accurately captures the dynamic changes in the signal, enhancing the real-time capabilities of spectrum sensing and effectively mitigating misjudgment issues in power-spectrum reconstruction caused by a lack of temporal information in complex electromagnetic environments.

### 3.1. Multi-Coset Sampling

**Figure 4:**
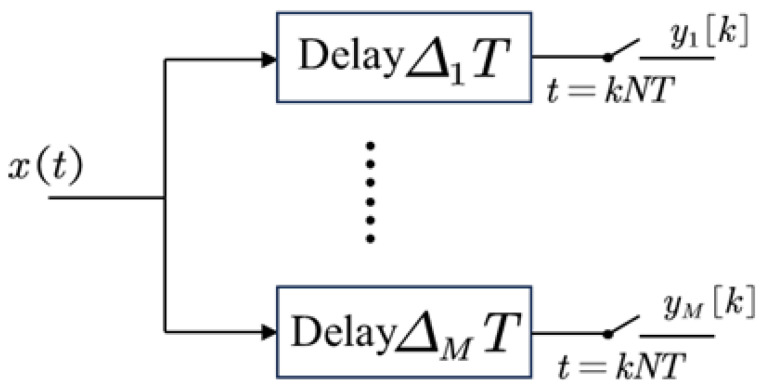
Architecture of Multi-Coset Sampling (MCS).

According to the statistical data presented in [4], the Multi-Coset Sampling (MCS) scheme requires the least computational effort and is also the simplest to implement, establishing it as the predominant compressed sampling structure currently available. MCS utilizes a periodic non-uniform sampling approach. In this framework, a continuous set of *L* sampling points is regarded as a single sampling block, within which *p* sampling points are selected, with p<L, maintaining a period of *L*, where *T* denotes the Nyquist sampling interval. During implementation, MCS is typically designed as a sampling structure that contains M channels, each responsible for processing a portion of the sampled data, as illustrated in Figure 4. According to compressive sensing theory, the parameter *M* must satisfy the theoretical lower bound M≥K, where *K* is the sparsity level of the signal, to ensure the Restricted Isometry Property (RIP) of the measurement matrix and guarantee unique reconstruction. However, in practical sparse signal recovery, moderate redundancy (e.g., M=1.5K∼2K) beyond the theoretical minimum is required to address non-ideal sparsity and noise interference.

In the $m^{th}$ channel, the signal is initially subjected to a fixed delay $\tau_{m}=\Delta_{m}T$. Subsequently, it is sampled by a low-speed analog-to-digital converter (ADC) at a sampling rate of NT, where *N* denotes the downsampling factor that represents the compression ratio of MCS relative to Nyquist sampling. A smaller value of *N* corresponds to higher resource utilization. The coefficient $\Delta_{m}$ is an integer less than N, and this set is given by

(30) $$\Delta =\underset{1\leq i\leq M}{\left\{\Delta_{i}\right\}}=\left\{\Delta_{1},\Delta_{2},\ldots ,\Delta_{M}\right\}$$

where

(31) $$0\leq \Delta_{1}<\Delta_{2}<\cdots <\Delta_{M}\leq L-1$$

When designing $\left\{\Delta \right\}$, for any integer *n* that satisfies $\left|n\right|\leq \left\lfloor \frac{N}{2}\right\rfloor$, there exist two indices $m_{1}$ and $m_{2}$ such that $n=\Delta_{m_{1}}-\Delta_{m_{2}}$, where ⌊·⌋ denotes the floor function. A detailed mathematical proof can be found in [17].

At the sampling moment *l*, the output of the $m^{th}$ branch is

(32) $$y_{m}\left[l\right]=x\left(lNT+\Delta_{m}T\right)=x\left[lN+\Delta_{m}\right]$$

For all channels’ sampling points, Equation (32) can be written in matrix form as

(33) $$y\left[l\right]=Cx\left[l\right]$$

where $C\in {\left\{0,1\right\}}^{M\times N}$ is a selection matrix with only one non-zero element per row. From the above analysis, it is evident that the sampling points of MCS constitute a subset of the Nyquist sampling points.

### 3.2. Fast Time–Frequency Reconstruction Algorithm

**Figure 5:**
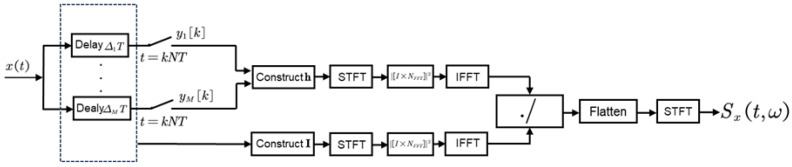
FTFR algorithm flowchart.

The overall flowchart of the FTFR algorithm is illustrated in Figure 5. First, the received signal $x\left(t\right)$ undergoes MCS to acquire sub-Nyquist samples. Next, utilizing the delay matrix Δ and the compressed sampling results $y\left[k\right]$, an indicator sequence $I\left[n\right]$ and data sequence $h\left[n\right]$ are constructed. Short-Time Fourier Transform (STFT) is applied to $I\left[n\right]$ and $h\left[n\right]$, respectively, to derive a matrix containing time–frequency information. Subsequently, the Inverse Fast Fourier Transform (IFFT) is computed row-wise for this matrix, and the elements at corresponding positions within the matrices are divided to compute the signal’s autocorrelation across different time windows. The resulting matrices, which encapsulate time–frequency information, are subsequently flattened after concatenation. Finally, STFT is applied to the flattened autocorrelation matrix to conduct time–frequency analysis. It is noteworthy that all operations depicted in the diagram are matrix operations, where ./ denotes element-wise division. A detailed derivation of the algorithm will be provided in the subsequent sections.

**Figure 6:**
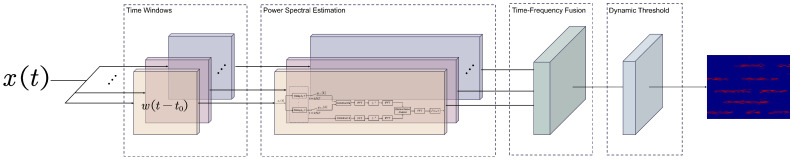
Explanatory diagram of FTFR algorithm.

To provide a clearer elucidation of the algorithmic process, Figure 5 is decomposed into a three-dimensional diagram as shown in Figure 6. The introduction of the STFT in place of the FFT fundamentally incorporates the use of a time window. Its primary objective is to integrate temporal information into the signal-processing framework. Theoretically, this implies that the signal undergoes initial processing through segmentation using time windows. When receiving the signal $x\left(t\right)$, the segment of the signal within each time window is represented by

(34) $$x_{i}\left(t\right)=x\left(t\right)\cdot w\left(t-t_{i}\right)$$

where $w\left(t\right)$ denotes the time window, and $t_{i}$ represents the center position of the $i^{th}$ time window. Each window has a width *W*, and it moves with a step size $H_{s}$, which is generally set to $H_{s}=W$. After applying MCS to the windowed signal, the output is

(35) $$\begin{array}{cc} y_{m,i}\left[l\right] & =x_{i}\left(lNT+\Delta_{m}T\right)\\ \; & =x\left(lNT+\Delta_{m}T\right)\cdot w\left(lNT+\Delta_{m}T-t_{i}\right) \end{array}$$

To establish the relationship between the sub-Nyquist sampled sequence and the Nyquist sampled sequence, we define the data sequence ${\left\{h_{i}\left[n\right]\right\}}_{n=0}^{LN-1}$ and indicator sequence ${\left\{I\left[n\right]\right\}}_{n=0}^{LN-1}$ within the $i^{th}$ time window as follows

(36) $$h_{i}\left[n\right]=\left\{\begin{array}{c} y_{m,i}\left[l\right],\;\;\;\;n=lN+\Delta_{m}\\ 0\;\;\;\;\;\;\;\;,\;\;\;\;\mathrm{otherwise} \end{array}\right.$$

(37) $$I\left[n\right]=\left\{\begin{array}{c} 1,\;\;\;\;n=lN+\Delta_{m}\\ 0,\;\;\;\;\mathrm{otherwise} \end{array}\right.$$

It is straightforward to determine that

(38) $$h_{i}\left[n\right]=x_{i}\left[n\right]\cdot I\left[n\right]$$

According to the Wiener–Khinchin theorem, the power-spectral density of a wide-sense stationary random process is given by the Fourier transform of its autocorrelation function. Therefore, the power spectrum within the $i^{th}$ window is

(39) $$P_{i}\left(\omega \right)=F\left[r_{x_{i}}\left[k\right]\right]$$

To achieve continuous time–frequency analysis, it is essential to fuse spectra from adjacent time windows, which can be accomplished through weighted averaging. Assuming that two adjacent time windows have the power spectrum $S_{x}\left(t_{i},\omega \right)$ and $S_{x}\left(t_{i+1},\omega \right)$, these can be fused using a weighting function $\alpha \left(t\right)$

(40) $$S_{x}\left(t,\omega \right)=\alpha \left(t\right)S_{x_{i}}\left(t_{i},\omega \right)+\left(1-\alpha \left(t\right)\right)S_{x_{i+1}}\left(t_{i+1},\omega \right)$$

where $\alpha \left(t\right)$ represents the transition relationship between $S_{x}\left(t_{i},\omega \right)$ and $S_{x}\left(t_{i+1},\omega \right)$. In this algorithm, the weighting function is characterized by a linear function, specifically $\alpha \left(t\right)=\frac{t-t_{i}}{t_{i+1}-t_{i}}$. By fusing the complete time–frequency representation of the signal according to the aforementioned method, the final time–frequency analysis formula is derived as

(41) $$S_{x}\left(t,\omega \right)=\sum_{i}\alpha_{i}\left(t\right)S_{x_{i}}\left(t_{i},\omega \right)$$

where $\alpha_{i}\left(t\right)$ is the weighting function for the $i^{th}$ time window.

At this point, our objective is to estimate the autocorrelation function $r_{x_{i}}\left[k\right]$ within the $i^{th}$ time window, for which we utilize the algorithm presented in [20].

(42) $$\begin{array}{cc} r_{x_{i}}\left[k\right] & =E\left[x_{i}\left[n\right]x_{i}*\left[n-k\right]\right]\\ \; & \approx \frac{1}{\left|Q_{k}\right|}\sum_{n\in Q_{k}}\left(x_{i}\left[n\right]x_{i}*\left[n-k\right]\right)\\ \; & \approx \frac{1}{Q_{k}}\sum_{n\in {\hat{Q}}_{k}}\left(x_{i}\left[n\right]x_{i}*\left[n-k\right]\right)\\ \; & =\frac{1}{Q_{k}}\sum_{n\in Q_{k}}\left(h_{i}\left[n\right]h_{i}*\left[n-k\right]\right)\\ \; & =\frac{r_{h_{i}}\left[k\right]}{Q_{k}} \end{array}$$

where $Q_{k}≜\left\{n\left|0\leq n-k\leq LN-1,0\leq n\leq LN-1\right.\right\}$, ${\hat{Q}}_{k}≜\left\{n\left|I\left[n\right]I\left[n-k\right]=1\right.\right\}$ and $Q_{k}≜\left|{\hat{Q}}_{k}\right|$. Here, $Q_{k}$ represents the set of valid indices for computing the autocorrelation at lag *k*, ${\hat{Q}}_{k}$ is the subset of those indices where both $I\left[n\right]$ and $I\left[n-k\right]$ are non-zero. The normalization factor $Q_{k}$ corresponds to the size of the set ${\hat{Q}}_{k}$.

First, we calculate $r_{h_{i}}\left[k\right]$, according to the definition of autocorrelation

(43) $$\begin{array}{cc} r_{h_{i}}\left[k\right] & =E\left[h_{i}\left[n\right]h_{i}*\left[n-k\right]\right]\\ \; & =\sum_{n\in Q_{k}}\left(h_{i}\left[n\right]h_{i}*\left[n-k\right]\right)\\ \; & =\sum_{n=0}^{LN-1}\left(h_{i}\left[n\right]h_{i}*\left[n-k\right]\right) \end{array}$$

To solve for $r_{h_{i}}\left[k\right]$, we define two new sequences ${\bar{h}}_{i}\left[n\right]$ and ${\hat{h}}_{i}\left[n\right]$,

(44) $${\bar{h}}_{i}\left[n\right]=\left\{\begin{array}{c} h_{i}\left[n\right],\;\;\;\;0\leq n\leq LN-1\\ 0\;\;\;\;\;\;,\;\;\;\;-LN-1\leq n<0 \end{array}\right.$$

(45) $${\hat{h}}_{i}\left[k\right]={\bar{h}}_{i}\left[-n\right]$$

Based on the convolution property, Equation (43) can be simplified to

(46) $$r_{h_{i}}\left[k\right]=\left({\bar{h}}_{i}⊛{\hat{h}}_{i}*\right)\left[k\right]$$

where ⊛ represents circular convolution. According to the circular convolution theorem, we obtain

(47) $$r_{h_{i}}=F_{2NL-1}^{-1}{\left|F_{2NL-1}\bar{h}\right|}^{2}$$

where $r_{h_{i}}≜{\left[r_{h_{i}}\left[-LN+1\right],\cdots ,r_{h_{i}}\left[LN+1\right]\right]}^{T}$ and ${\bar{h}}_{i}≜{\left[{\bar{h}}_{i}\left[-LN+1\right],\cdots ,{\bar{h}}_{i}\left[LN+1\right]\right]}^{T}$. $F_{2NL-1}$ and $F_{2NL-1}^{-1}$ denote the FFT and IFFT of $\left(2NL-1\right)$ points, respectively.

Subsequently, we calculate $Q_{k}$,which is defined as

(48) $$Q_{k}=\sum_{n\in Q_{k}}I\left[n\right]I*\left[n-k\right]$$

Similarly, we define two new sequences $\bar{I}\left[n\right]$ and $\hat{I}\left[n\right]$; that is,

(49) $$\bar{I}\left[n\right]=\left\{\begin{array}{c} I\left[n\right],\;\;0\leq n\leq LN-1\\ 0\;\;\;\;,\;\;-LN-1\leq n<0 \end{array}\right.$$

(50) $$\hat{I}\left[n\right]=\bar{I}\left[-n\right]$$

By analogy, based on the convolution property, Equation (48) can also be simplified to

(51) $$I\left[k\right]=\left(\bar{I}⊛\hat{I}*\right)\left[k\right]$$

According to the circular convolution theorem, we obtain

(52) $$q=F_{2NL-1}^{-1}{\left|F_{2NL-1}\bar{I}\right|}^{2}$$

where $q≜{\left[Q_{-LN+1},\cdots ,Q_{LN-1}\right]}^{T}$ and $\bar{I}≜{\left[\bar{I}\left[-LN+1\right],\cdots ,\bar{I}\left[LN-1\right]\right]}^{T}$.

To perform time–frequency analysis, we first compute the autocorrelation function of the signal within each time window, as described in Equation (42). Subsequently, based on the Wiener–Khinchin theorem, we derive the power spectrum for each time window from the computed autocorrelation functions. Finally, by applying Equation (41), we obtain a continuous time–frequency analysis result through the weighted fusion of power-spectral information from each time window. The entire algorithm process is summarized and presented in Algorithm 1.

### 3.3. Computational Complexity

The FTFR algorithm proposed in this paper swiftly reconstructs the power spectrum within each time window by applying a width-*W* time window to the signal $x\left(t\right)$, followed by the fusion of the final time–frequency information. Assuming that the total signal length is *K*, and that each window moves with a step size $H_{s}$, a total of $I=\lceil \frac{K-W}{H_{s}}\rceil +1$ windows are required, where ⌈ ⌉ denotes the ceiling function for rounding up. It can be observed that within each time window, the computations mainly involve simple multiplication and division operations as well as FFT/IFFT operations, while the final time–frequency data-fusion stage entails only weighted averaging.
**Algorithm 1** Fast time–frequency reconstruction algorithm**Require:**
  signal $x\left(t\right)$, time window $w\left(t\right)$, window width *W*, window center position $t_{i}$, sub-sampling factor *N*, sampling block length *L***Ensure:**
  Time–frequency representation $P_{x}\left(t,\omega \right)$ of the signal $x\left(t\right)$1:Initialize an empty array of signal segments Segments[]2:**for** each time window $w\left(t-t_{i}\right)$ **do**3:    Extract signal segment $x_{i}\left(t\right)$ from $x\left(t\right)$, centered at window center $t_{i}$ with width *W*4:    Segment $x_{i}\left(t\right)$ and append each segment to Segments[]5:**end for**6:Initialize arrays for sub-Nyquist sampling sequence $\left\{y\left[n\right]\right\}$ and Nyquist sampling sequence $\left\{x\left[n\right]\right\}$7:**for** each signal segment $x_{i}\left(t\right)$ in Segments[] **do**8:    Apply Multi-Coset Sampling (MCS) to obtain sub-Nyquist sampling output $y_{m,i}\left[l\right]$9:    Define ${\bar{h}}_{i}\left[n\right]$ and $\bar{I}\left[n\right]$10:**end for**11:Initialize an empty array for power spectra P[]12:**for** each time window $w\left(t-t_{i}\right)$ **do**13:    Calculate the autocorrelation $r_{x_{i}}$ for the *i*-th time window14:    Use autocorrelation estimation and Fast Fourier Transform (FFT) to derive $P_{x_{i}}\left(\omega \right)$15:    Append $P_{x_{i}}\left(\omega \right)$ to P[]16:**end for**17:Initialize the time–frequency representation TFR18:**for** adjacent time windows *i* and i+1 **do**19:    Fuse $P_{x_{i}}$ and $P_{i+1}$ using a weighting function20:    Update TFR to include the fused power spectrum21:**end for**22:**return** the final time–frequency representation TFR of the signal

Traditional time–frequency reconstruction algorithms based on compressive sensing typically employ the OMP algorithm, with a single iteration complexity of $O\left(DO\right)$, where *D* is the signal dimension and *O* is the number of dictionary atoms. For *I* time windows, the total complexity becomes $O\left(IDO^{2}\right)$. Classic covariance-sensing-based methods avoid sparse recovery but require matrix inversion for autocorrelation reconstruction, with a per-window complexity of $O\left(L^{3}\right)$, where *L* is the correlation matrix dimension), leading to a total complexity of $O\left(IL^{3}\right)$ for *I* windows. In contrast, FTFR reduces computational complexity from polynomial to logarithmic operations via FFT/IFFT, also lowering ADC sampling rate requirements. Specifically, to compute the power spectrum of the signal $x_{i}\left(t\right)$ within each time window at a spectral resolution of $\frac{1}{\left(2NW-1\right)T}$, it is necessary to calculate the correlation function $r_{h_{i}}$ and the normalization factor q, followed by performing a $\left(2NL-1\right)$-points FFT. Consequently, the complexity within each time window is

(53) $$O\left(\left(6WN-3\right)\log\left(2WN-1\right)+2WN-1\right)$$

The overall complexity for calculating the power spectrum across all time windows is

(54) $$O\left(I\cdot \left[\left(6WN-3\right)\log\left(2WN-1\right)+2WN-1\right]+\left(I-1\right)W\right)$$

During the time–frequency information fusion phase, the weighted fusion of adjacent time windows requires weighted averaging calculations for each frequency point, resulting in a complexity of $O\left(\left(I-1\right)W\right)$. By combining both aspects, the overall complexity of the FTFR algorithm is

(55) $$O\left(I\cdot \left[\left(6WN-3\right)\log\left(2WN-1\right)+2WN-1\right]+\left(I-1\right)W\right)$$

Since the FFT operations constitute the primary component of the computation, they can be executed in parallel efficiently, rendering this method particularly suitable for real-time applications. Through optimized implementation on high-performance hardware, such as Field-Programmable Gate Arrays (FPGAs), the reconstruction of the time–frequency map can be achieved in a short timeframe. Currently, Intel’s Agilex 7 FPGA can perform 38 trillion floating-point operations per second, enabling it to generate the time–frequency map for a bandwidth of 1 GHz sampled at a spectral resolution of 50 kHz in approximately 644 microseconds, which effectively meets the demands of most real-time sensing applications.

## 4. Simulation Results

In this section, we assess the performance of the proposed FTFR algorithm through a series of simulation experiments. To test and validate the fundamental performance characteristics of the algorithm, we generated broadband stationary signals for the experiments. Specifically, in our experiments, we processed zero-mean, unit-variance Gaussian white noise using a Finite Impulse Response (FIR) filter to generate five wide-sense stationary signals within the frequency range of [0, 500] MHz. The carrier frequencies of these signals are [55, 135, 220, 315, 420] MHz, respectively, each with a bandwidth of 15 MHz. According to the Nyquist sampling theorem, the sampling frequency must be at least $f_{nyq}=1\;\mathrm{GHz}$. Our objective is to determine the presence of these signals at various moments and frequency positions; therefore, we collected data samples over a sampling duration of 1 s, with the duration of signals on different carriers constrained as follows:

$$\left\{T_{55\;\;\mathrm{MHz}}=\left[0,0.3\right]\cup \left[0.45,0.6\right]\cup \left[0.7,1.0\right],\right.$$

$$\;T_{135\;\;\mathrm{MHz}}=\left[0.2,0.7\right],$$

$$\;T_{220\;\;\mathrm{MHz}}=\left[0.1,0.5\right]\cup \left[0.6,0.9\right],$$

$$\;T_{315\;\;\mathrm{MHz}}=\left[0,0.25\right]\cup \left[0.4,0.8\right],$$

$$\;\left.T_{420\;\;\mathrm{MHz}}=\left[0.3,0.65\right]\cup \left[0.8,1.0\right]\right\}s$$

**Figure 7:**
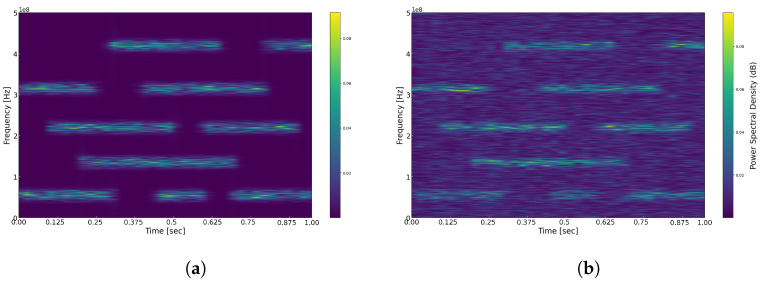
(**a**) Time–frequency spectrum of the original signal with Hamming window and width 1024. (**b**) Time–frequency spectrum of the original signal with AWGN with Hamming window and width 1024.

The original time–frequency representation of the simulated signals is illustrated in Figure 7a. We employed a Hamming window as the analysis window, selecting a width of 1024 to balance time resolution and frequency resolution. To implement the FTFR algorithm, Multi-Coset Sampling (MCS) was initially employed to obtain sub-Nyquist samples. In this arrangement, we utilized 8 channels, specifically M=8, with each channel’s sampling rate set at 60 MHz and different delays $\Delta =\left\{1,2,3,4,6,8,9,12\right\}$, which satisfy the requirements outlined in [17]. The frequency resolution was established at 50 kHz, corresponding to a power-spectrum length of 20,000. To achieve this resolution, each sampling channel was required to collect at least $L\geq \frac{20,000}{2N}\approx 589$ samples. To more closely simulate real-world scenarios, we added noise to the signals. Figure 7b illustrates the time–frequency representation with a Signal-to-Noise Ratio (SNR) set at 0 dB. Unless otherwise specified, subsequent simulations will also use an SNR=0 dB. After applying the FTFR algorithm to reconstruct the time–frequency analysis, we set a threshold to clearly identify spectral holes based on the signal’s power-spectrum characteristics, i.e.,

(56) $$\gamma =k*E\left[P_{x_{i}}\left(\omega \right)\right]$$

where *k* is a constant less than 1 used to adjust the sensitivity of the threshold, and $E\left[P_{x_{i}}\left(\omega \right)\right]$ denotes the average value of the power spectrum.

**Figure 8:**
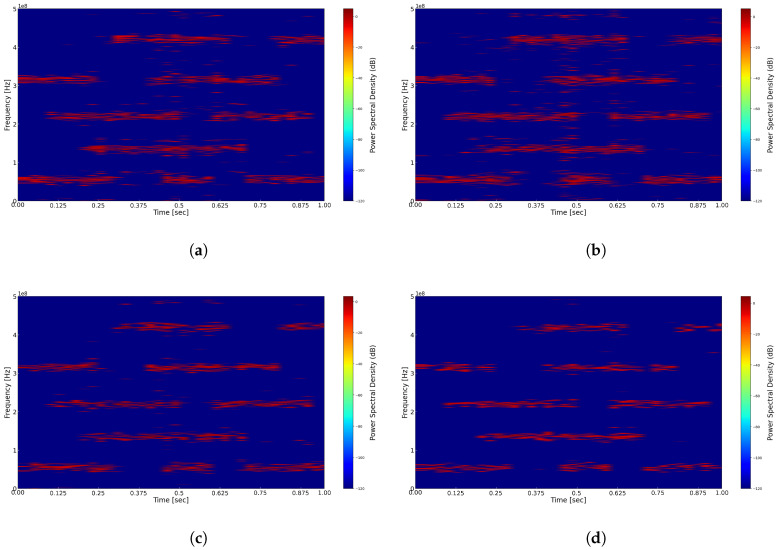
(**a**) Reconstructed time–frequency spectrum with Hann window. (**b**) Reconstructed time–frequency spectrum with Hamming window. (**c**) Reconstructed time–frequency spectrum with Blackman window. (**d**) Reconstructed time–frequency spectrum with Gaussian window.

We first compare and analyze the results of reconstructing the time–frequency representation using different window functions, with the fixed window width set to 1024, including the application of a threshold. As illustrated in Figure 8, experiments were conducted using the Hann window, Hamming window, Blackman window, and Gaussian window. From the figures, it is evident that in the time–frequency representations of Figure 8a Hann window, Figure 8b Hamming window, and Figure 8c Blackman window, the energy distribution of signals across different carriers appears relatively blurred, resulting in lower time and frequency resolutions. Conversely, in the time–frequency representation of Figure 8d Gaussian window, the signal’s energy distribution is clearer, yielding higher time and frequency resolutions. This difference may be attributed to the wider main lobes of the Hann, Hamming, and Blackman windows, which result in lower time resolution and a reduced ability to accurately capture rapid changes in the signal. In contrast, the Gaussian window offers improved localization in both the time and frequency domains, thereby simultaneously providing superior time and frequency resolutions. It is important to note that the computational complexity of the Gaussian window is higher than that of other windows, and it requires the adjustment of the shape parameter and standard deviation to achieve optimal performance. Relevant simulation experiments will be discussed in detail in future research.

**Figure 9:**
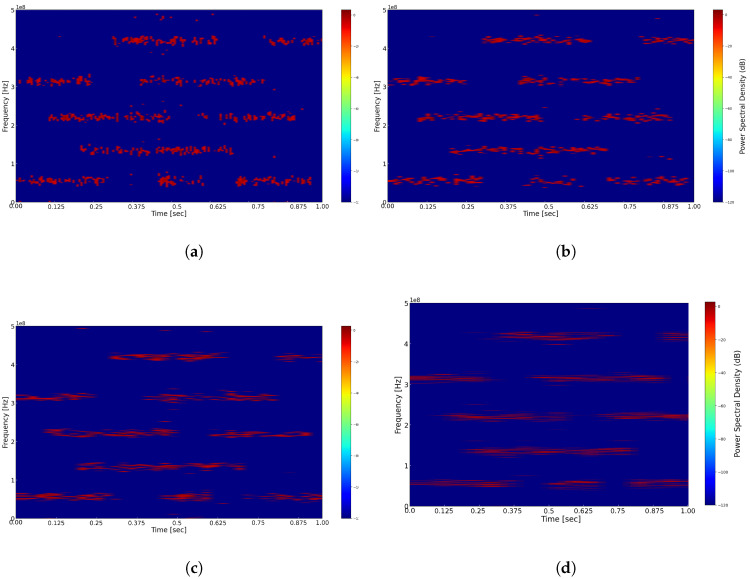
(**a**) Time–frequency reconstruction with width 256. (**b**) Time–frequency reconstruction with width 512. (**c**) Time–frequency reconstruction with width 1024. (**d**) Time–frequency reconstruction with width 2048.

The width of the window function plays a crucial role in time–frequency analysis, directly influencing the resolution of the signal in both the time and frequency domains. According to the Uncertainty Principle, it is impossible to simultaneously achieve high time resolution and high frequency resolution in time–frequency analysis. Consequently, we further investigate the effect of various time-window widths on the reconstruction of the signal’s time–frequency representation. As illustrated in Figure 9, panels (a–d) depict the reconstructed time–frequency representations after applying a threshold for window widths of 256, 512, 1024, and 2048, respectively. A window width of 256 offers high time resolution, allowing for the capture of short-term dynamic changes in the signal; however, it results in lower frequency resolution, which makes it challenging to accurately distinguish between signals with close frequencies, potentially leading to misjudgments of spectral holes. Conversely, a time-window width of 2048 provides higher frequency resolution; however, this comes at the expense of time resolution, risking the oversight of many briefly appearing spectral holes. In comparison, window widths of 512 and 1024 strike a better balance between frequency and time resolution. Considering the application context of spectrum sensing in TDCS, these window widths enable the precise and efficient identification of spectral holes.

**Figure 10:**
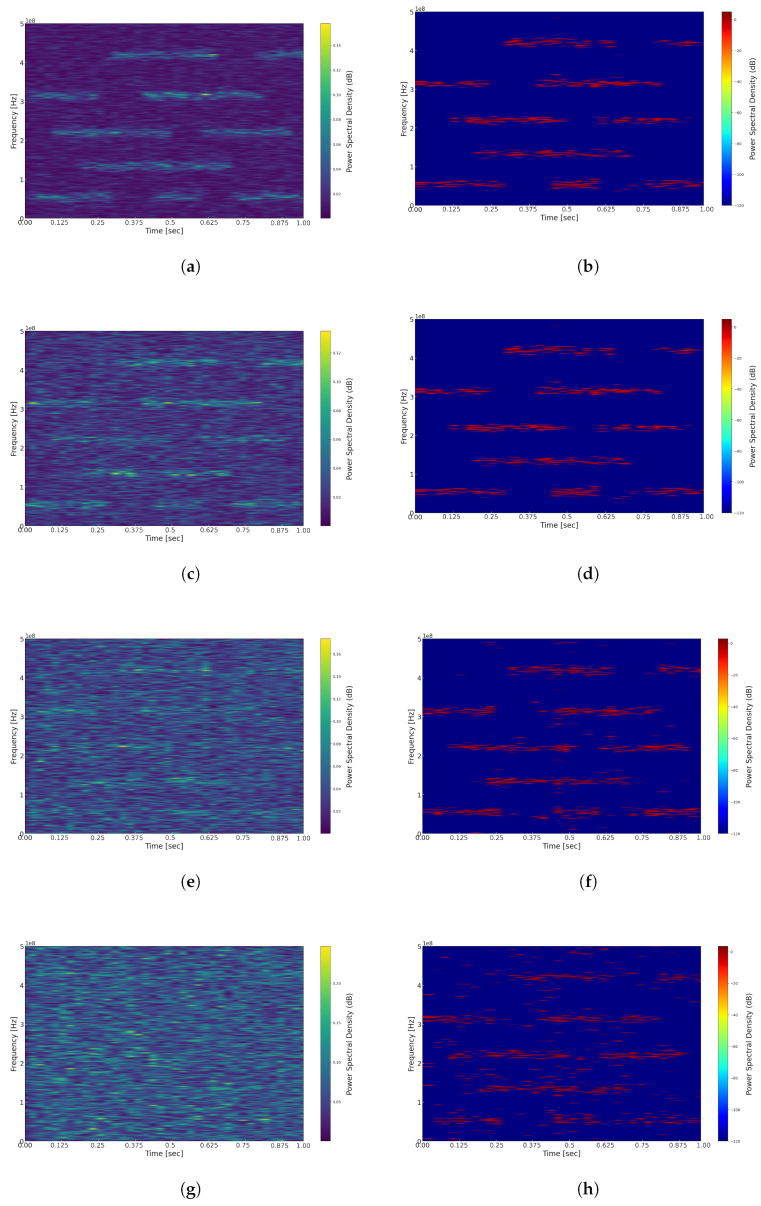
(**a**) Reconstructed spectrum corresponding to SNR = 0 dB. (**b**) Reconstructed spectrum corresponding to SNR = 0 dB. (**c**) Time–frequency spectrum of signal with SNR = −5 dB. (**d**) Reconstructed spectrum corresponding to SNR = −5 dB. (**e**) Time–frequency spectrum of signal with SNR = −10 dB. (**f**) Reconstructed spectrum corresponding to SNR = −10 dB. (**g**) Time–frequency spectrum of signal with SNR = −15 dB. (**h**) Reconstructed spectrum corresponding to SNR = −15 dB.

To comprehensively validate the performance of the FTFR algorithm, we compare the time–frequency reconstruction results under different SNR conditions in Figure 10. An analysis of this figure reveals that at SNR=0 dB, the FTFR algorithm can effectively restore the time and frequency distribution of the signals. As the SNR decreases, the time–frequency representation of the original signal gradually becomes affected by noise, resulting in less distinct signal characteristics. Nonetheless, the FTFR algorithm remains capable of relatively clear reconstruction of the primary time and frequency distributions. When the SNR further drops to −15 dB, the original signal is nearly overwhelmed by noise, resulting in increased interference in the reconstructed time–frequency representation. Despite this, it is still possible to approximately reconstruct the main time and frequency distributions. Therefore, the FTFR algorithm proposed in this paper can achieve satisfactory reconstruction results even under low SNR conditions. Nonetheless, further improvements are necessary to filter out additional noise for improved performance.

In order to further evaluate the performance of the proposed fast time–frequency reconstruction (FTFR) algorithm under various Signal-to-Noise Ratio (SNR) conditions, we have plotted Receiver Operating Characteristic (ROC) curves. True-Positive Rate (TPR) and False-Positive Rate (FPR) are key metrics for evaluating detection performance, defined in this context as follows:

• True-Positive Rate (TPR): The proportion of actually occupied frequency bands that are correctly identified as occupied by the FTFR algorithm. It is calculated as $TPR=\frac{TP}{TP+FN}$, where TP represents true positives, i.e., the number of frequency bands that are actually occupied and correctly detected; FN represents false negatives, i.e., the number of frequency bands that are actually occupied but not detected.
• False-Positive Rate (FPR): The proportion of actually unoccupied frequency bands that are incorrectly identified as occupied by the FTFR algorithm. It is calculated as $FPR=\frac{FP}{FP+TN}$, where FP represents false positives, i.e., the number of frequency bands that are actually unoccupied but incorrectly marked as occupied by the algorithm; TN represents true negatives, i.e., the number of frequency bands that are actually unoccupied and correctly identified as such.

**Figure 11:**
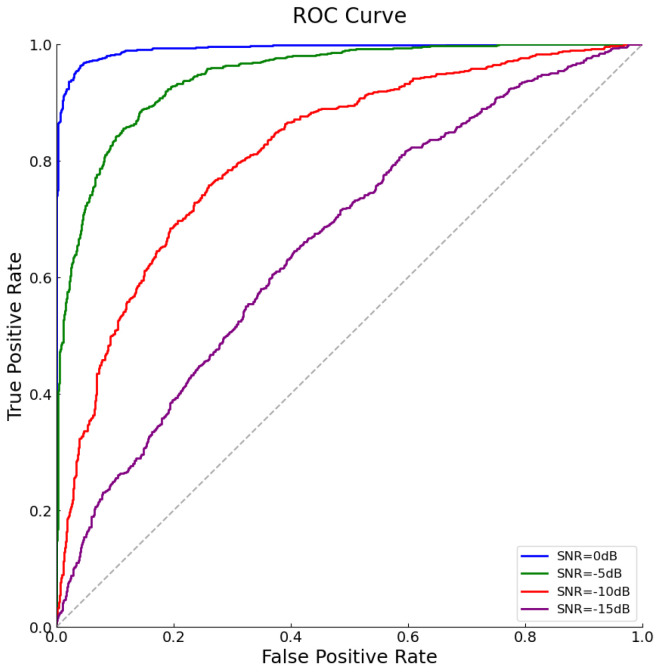
ROC curves under different SNRs.

As shown in Figure 11. These curves illustrate the detection performance at SNR levels of 0 dB, −5 dB, −10 dB, and −15 dB. In Figure 11, as the SNR decreases from 0 dB to purple −15 dB, we observe a shift towards the lower left corner, indicating that the balance between TPR and FPR deteriorates with increasing noise levels, showing a decrease in signal detection accuracy. Notably, even under challenging low SNR conditions, such as −15 dB, the FTFR algorithm maintains a certain level of detection capability, which can be attributed to its effective noise-reduction capabilities and accurate capture of signal characteristics. Additionally, by analyzing the Area Under the Curve (AUC) of the ROC curves, one can quantify the overall performance of the algorithm at different SNR levels. The closer the AUC value is to 1, the better the discriminative ability of the algorithm. In this study, even at the lowest SNR level, the FTFR algorithm demonstrates relatively high AUC values, proving its good applicability in complex electromagnetic environments.

**Figure 12:**
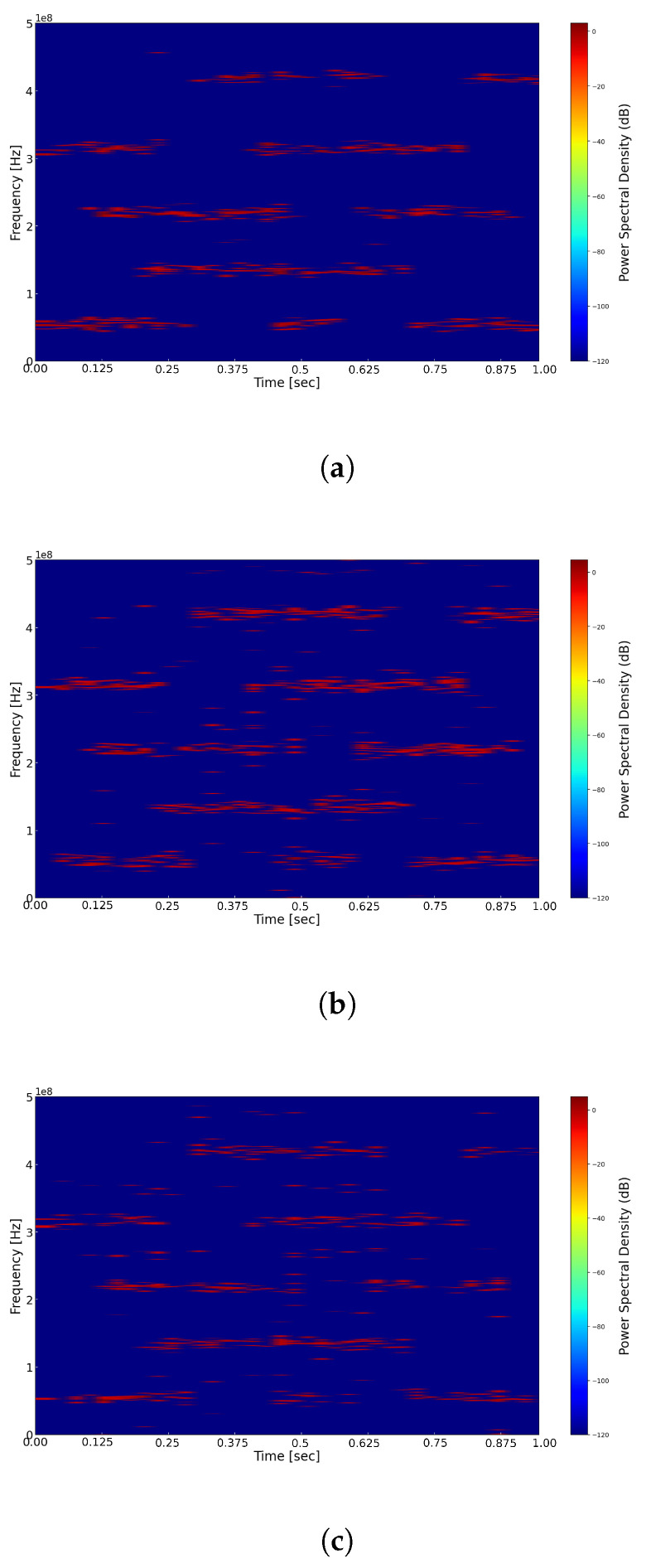
(**a**) Reconstructed time–frequency spectrum with ADC sampling rate of 100 MHz. (**b**) Reconstructed time–frequency spectrum with ADC sampling rate of 60 MHz. (**c**) Reconstructed time–frequency spectrum with ADC sampling rate of 50 MHz.

Subsequently, we investigated the impact of different down-sampling factors *N* on the time–frequency reconstruction performance under an SNR=0. In the experiment, we set the ADC sampling rates to 100 MHz, 60 MHz, and 50 MHz, corresponding to down-sampling factors *N* of 10, 17, and 20, with compression ratios of 0.8, 0.48, and 0.4, respectively. We present the reconstructed time–frequency representations at different sampling rates in Figure 12. From this figure, it is evident that as the sampling rate increases, the performance of time–frequency reconstruction also improves. Higher sampling rates facilitate a more accurate capture of the finer details of the signal, thereby enhancing the quality of the reconstruction.

**Figure 13:**
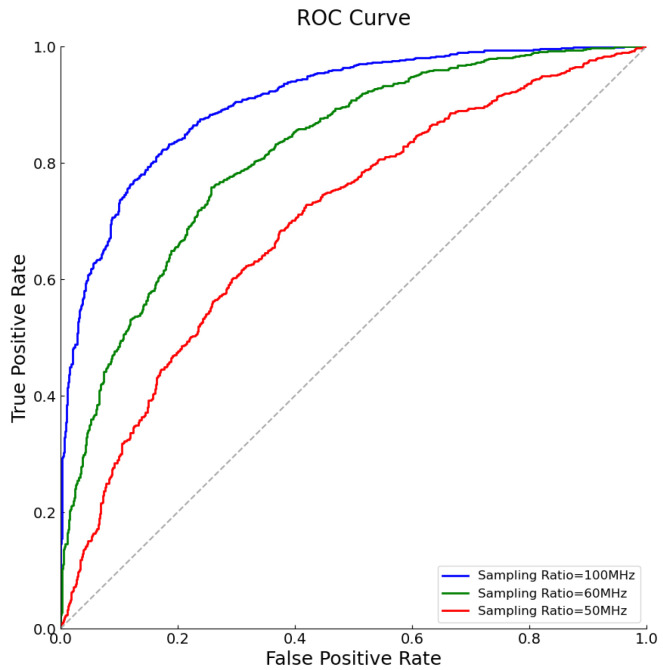
ROC curves under different sampling ratios at SNR = −10 dB.

We went on to analyze the ROC curves corresponding to different sampling rates, as shown in Figure 13. At this noise level, with the increase in sampling rate, the balance between TPR and FPR gradually shifts towards the upper right, indicating that a higher sampling rate contributes to improved signal-detection accuracy. Specifically, at a 100 MHz sampling rate, the ROC curve is closest to the top left corner, demonstrating the highest discrimination and optimal detection performance; whereas at a 50 MHz sampling rate, the position of the ROC curve is lower, reflecting relatively weaker detection capability. By comprehensively analyzing Figure 12 and Figure 13, although a higher sampling rate can achieve better performance, increasing the sampling rate to improve reconstruction quality comes with certain costs. On one hand, data volume increases with the rise in sampling rate, which not only raises storage and transmission costs but may also lead to increased processing delays. On the other hand, higher sampling rates require more advanced hardware capabilities, including greater computational power and faster data-processing speeds. Therefore, in practical applications, it is necessary to weigh the selection of sampling rates based on specific needs to achieve the best balance between performance and cost.

**Figure 14:**
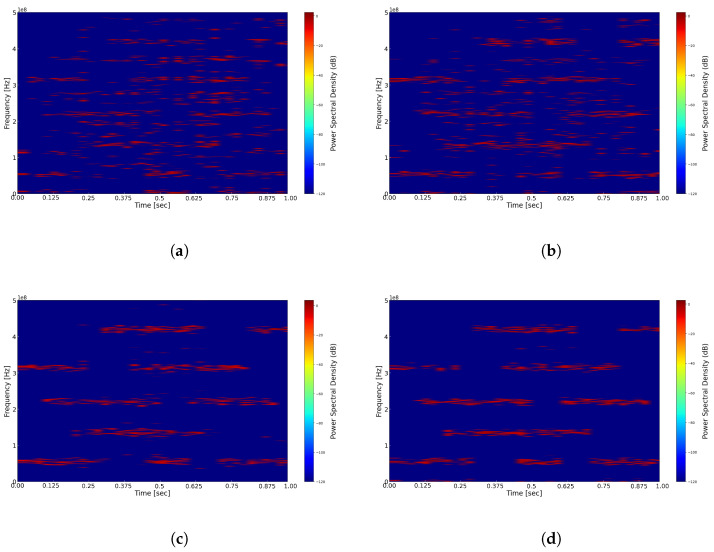
(**a**) Time –frequency reconstruction with M = 5. (**b**) Time–frequency reconstruction with M = 6. (**c**) Time–frequency reconstruction with M = 8. (**d**) Time–frequency reconstruction with M = 10.

Finally, we compare the effect of different MCS compression channel number *M* on the reconstruction results, as shown in the Figure 14. The values of M corresponding to (a–d) are 5, 6, 8, and 10, respectively. The results show that as *M* increases, the details in the time–frequency representation become richer, and the dynamic changes of the signal are captured more clearly. Specifically, when M=5, that is, it is in a critical state, the time–frequency representation is relatively blurry, making it difficult to discern the details and dynamic changes of the signal. When M=6, the details in the time–frequency representation improve, but there is still some blurriness, and the dynamic changes of the signal are not clear enough. When M=8, the time–frequency representation is very detailed, with clear dynamic changes of the signal, allowing for accurate capture of the time–frequency characteristics. When M=10, the details in the time–frequency representation further increase, but the improvement over M=8 is not significant, and the computational complexity and data volume also increase. From the previous complexity analysis, although M=10 can achieve slightly better results than M=8, it also brings higher computational complexity. Overall, M=8 is a good choice as it provides excellent performance in capturing the details and dynamic changes in the time–frequency representation while maintaining lower computational complexity and data volume.

## 5. Conclusions

In this paper, we address the challenge of time–frequency reconstruction in WBSS by proposing a FTFR algorithm. The objective of this algorithm is to efficiently and accurately recover the time–frequency characteristics of signals from sub-Nyquist samples, thereby facilitating the dynamic selection of spectra in TDCS. By incorporating the MCS structure, the FTFR algorithm acquires sub-Nyquist samples, significantly reducing the required sampling rate. By leveraging low-complexity operations such as STFT, IFFT, and matrix computations, the algorithm achieves precise calculation of the autocorrelation for signals across different windows. Through the fusion of spectra from adjacent time windows, the algorithm effectively captures the dynamic changes in the signals. The proposed algorithm demonstrates lower computational complexity compared to previous methods for reconstructing time–frequency analyses, rendering it suitable for practical applications. A series of simulation experiments validate the computational efficiency and effectiveness of the algorithm, indicating its capacity to approximate the primary time and frequency distributions even under low SNR conditions.

However, during the time–frequency fusion phase, this study primarily considered fusion along the temporal dimension, excluding the incorporation of phase information. If a discontinuity occurs in the phase during the fusion process, it may introduce noise into the frequency spectrum. This limitation may result in misjudgments in the reconstructed time–frequency representation, potentially affecting the accurate capture of transient signal features. In future research, we will concentrate on improving the time–frequency fusion strategy and exploring methods to incorporate phase information, further enhancing the accuracy and reliability of the time–frequency representation.

## Data Availability

The data used in this study were generated through simulations, and the specific parameters and generation methods are detailed in the article. If needed, readers can reproduce the data based on the parameters provided in the manuscript.

## Abbreviations

The following abbreviations are used in this manuscript:

CR	Cognitive Radio
TDCS	Transform Domain Communication Systems
WBSS	Wideband Spectrum Sensing
FTFR	Fast Time–Frequency Reconstruction
SNR	Signal-to-Noise Ratio
NBSS	Narrowband Spectrum Sensing
ADC	Analog-to-Digital Converter
CS	Compressed Sensing
CCS	Compressed Covariance Sensing
AIC	Analog-to-Information Converter
MWC	Modulated Wideband Converter
MCS	Multi-Coset Sampling
BP	Basis Pursuit
OMP	Orthogonal Matching Pursuit
CoSaMP	Compressive Sampling Matching Pursuit
SOMP	Simultaneous Orthogonal Matching Pursuit
FFT	Fast Fourier Transform
DFT	Discrete Fourier Transform
STFT	Short-Time Fourier Transform
IFFT	Inverse Fast Fourier Transform
FPGA	Field-Programmable Gate Array
FIR	Finite Impulse Response
AWGN	Additive white Gaussian noise
